# Parallel between Cerebral Fever and Worm Fever

**Published:** 1826-07

**Authors:** 


### PARALLEL BETWEEN THE SYMPTOMS OF THE CEREBRAL FEVER AND WORM FEVER OF CHILDREN. BY M. GINTRAE. PHYSICIAN OF BORDEAUX.

[Journal Général de Médecine, 1825.]

The similarity, or at least the analogy, of cerebral irritation and worm affections, or more properly speaking, intestinal affections, in children, has deceived the most attentive physicians, and is hourly leading a great many practitioners into erroneous decisions and improper practice, in every country of Europe. How common is it to hear a man speak of the number of cases of acute hydrocephalus or brain fever he has had to treat, and the great many cures he has performed of such; when, in reality, three fourths of these cases were infantile remittent, or intestinal fever. The following parallel, though far from being free from objections, may be of some use to the young practitioner at the bed-side of sickness. It may be premised that there is no one pathological symptom which can be depended on as characterising idiopathic cerebral fever, nor yet the intestinal. We must draw our conclusions from the whole of the symptoms taken collectively.

1. Those children who are most disposed to worm or intestinal affections, are of the lymphatic temperament, weak constitution, and lax fibre. Those most disposed to cerebral irritations, on the contrary, are the robust, the active, the irritable, and of the sanguine temperament.

2. The *former* have large bellies, and eat much. The *latter* have large heads, and the facial angle near the 90th degree.

3. Children who have previously had worms or intestinal affections, are more disposed to have the same again, and, therefore, their previous history should be carefully enquired into. On the other hand, there is but too much reason to believe that the disposition to cerebral irritation is often hereditary, and, therefore, the history of the family is deserving of investigation.

4. Female children are supposed to be more frequently affected with worms, and males with cerebral fever.

5. This last (cerebral) affection often results from external causes, as blows on the head, falls, insolation, suppression of discharges from the neighbourhood of the head, or of cutaneous eruptions. The production of worms and of intestinal affections, is generally facilitated by all debilitating causes, bad diet, too much vegetables, pastry, salted viands, and improper drink, to which may be added, too much medicine.

6. In cerebral fever we may have pain in the belly; but where there are worms in the primæ viæ, this pain is much greater, and especially when the stomach is empty. It is relieved by the ingestion of food.

7. Vomiting is a common symptom at the commencement of cerebral fever—it rarely takes place in worm affections, unless these animals ascend into the stomach, and then they are often discharged during vomiting.

8. In cerebral affections the appetite is impaired or annihilated—in worm affections it is commonly augmented.

9. In idiopathic cerebral affection the abdomen becomes flattened. Dr. Golis has particularly insisted on the importance of this symptom. Where worms are the cause of the fever, the belly is hard and distended—borborygmi are heard, and there are eructations.

10. Constipation is almost always an attendant on idiopathic cerebral fever, and when the motions appear, they are disordered, generally green, or slimy, or gellatinous. Worms, on the other hand, generally keep up more or less of diarrhœa, the motions being mucous, glairy, and fetid.

11. In worm affections the secretion of bile is increased. Brera regards this sign as very remarkable. It seldom obtains in cerebral affections, where the mouth is generally dry.

12. Cerebral irritations produce, in the beginning, redness at the tip, and along the edges of the tongue—worms, on the contrary, cause the root and middle of the tongue to be covered with a thick mucous fur.

13. The breath is fetid in worm affections, but rarely so in cerebral.

14. Cephalalgia is an almost constant symptom of cerebral affection. It is acute, and often causes the child to cry out, *oh, my head!* In verminous fever, the pain never arrives at this height. It is vague, obtuse, and the child seldom complains of it in particular.

15. In cerebral affections, the child often directs his hand to his head—while in verminous diseases, it is more commonly to the nose that the fingers are directed, in consequence of the itching there.

16. In both kinds of affection, the sleep is occasionally disturbed—but where the brain is the seat of lesion, the sleep is never natural—it is a kind of drowsiness, amidst which the moanings of the child are frequently heard—in worm affections the sleep is profound, though often interrupted by dreams and startings.

17. In both affections we observe grindings of the teeth; but when there are worms, we will often perceive a movement of deglutition during sleep, hiccup, and occasionally certain convulsive movements of the thumb and index finger.

18. The convulsions which we see both in cerebral and worm affections, may be very severe in either case, and greatly resemble each other; but in the *former* they are generally preceded by pain in the head, drowsiness, fever, &c. whereas, in the *latter* class, the convulsions are rarely ushered in by the symptoms abovementioned.

19. The coma which we occasionally observe in verminous affections, comes on very suddenly; but does not last long, and often leaves no trace of its existence.

20. The delirium, in cerebral fever, is very rarely violent—that produced by worms, occasions more agitation, and more extravagant actions.

21. The paralyses that occur are always more serious and permanent in the idiopathic cerebral maladies—more partial, transient, and variable in the *verminous complaints.*

22. Dilatation of the pupils often takes place in the *latter* class, and even before the attack of illness, in which case there is no aversion to the light—no affection of the sight. In the early period of cerebral irritation, the eye cannot bear light—the pupils are often contracted—and the dilatation that succeeds is only the result of loss of sensibility in the retina. In these cases there will be perceived an oscillatory movement in the iris, when a lighted candle is brought near the eye, and which M. Odier of Geneva, considers as an indication of effusion into the ventricles.

23. Strabismus is a strong symptom of cerebral lesion, especially of compression—it is rarely observed in verminous diseases.

24. In children affected with worms there is generally seen a dark circle round the eyes—a symptom but seldom observed in cerebral affections. In these *last* the nostrils are dry—in *worm fever* they are moistened with mucous matters. In the *latter* there is often a puffy swelling of the upper lip, the same as is seen in scrofulous children. It is rarely the case in idiopathic cerebral fever.

25. The complexion in worm cases is pale and leaden—in cerebral affections it is very variable, sometimes pale, but more commonly flushed.

26. Rolling the head on the pillow is a sign of cerebral, rather than of worm affection.

27. The pulse, as was before observed, presents, in cerebral irritation, great inequality, as, first frequency, then slowness, and then again great quickness. These modifications are not distinguishable in verminous affections. In these, the pulse is generally small, unequal, and occasionally intermittent.

28. M. Cruvelheir has always observed the respiration unequal in hydrocephalic affections, a symptom which he considers as pathognomonic—but this phenomenon occurs often in other affections of children.

29. The temperature of the skin is elevated in cerebral fever, but in worm-fever it is little above the natural level. In the *former* the heat of the head is much above that of the abdomen—while the reverse is the case in the *latter.* It is on this account that children with worm-fever always feel better after taking cold drink. The skin is also drier in cerebral, than in verminous affections.

30. The emaciation in cerebral fever is very rapid and general—in verminous disease, there is also marasmus, but it is not near so rapid. It is particularly observable in the extremities, while the abdomen preserves its size.

31. In cerebral fever the urine is very scanty, red, and sedimentous. In worm fever, the urine is sometimes clear and plentiful—more frequently troubled like whey, and letting fall a whitish sediment.

32. In verminous affections, there is much instability in the symptoms—thus coma, delirium, blindness, aphonia, &c. succeed each other with rapidity:—while, in cerebral fevers, we find a greater obstinacy —a more sustained march of the symptoms, which are regularly progressive.

The foregoing parallel or contrast, call it which we may, appears to be drawn from the observations of the best practical writers on the two diseases, and is well deserving of attention from the young practitioner. It must be remarked, however, that whatever be the original seat of the disease, when the head becomes affected, even sympathetically, we must attend carefully to that feature of the complaint. Nor does this part of the treatment interfere with that which is properly directed to the expulsion of worms, or the removal of bad secretions from the primæ viæ. These last very frequently determine cerebral irritation, in certain constitutions, and lead to hydrocephalic effusion in the end.

